#  The N-terminal polypeptide derived from vMIP-II exerts its anti-tumor activity in human breast cancer by regulating lncRNA SPRY4-IT1

**DOI:** 10.1042/BSR20180411

**Published:** 2018-10-17

**Authors:** Haihua Wu, Yueyue Wang, Tiantian Chen, Yu Li, Haifeng Wang, Lingyu Zhang, Sulian Chen, Wenrui Wang, Qingling Yang, Changjie Chen

**Affiliations:** 1 Anhui Province Key Laboratory of Translational Cancer Research, Bengbu Medical College, Bengbu, Anhui, 233030, China; 2 Xuzhou Central Hospital, Xuzhou, Jiangsu, 221000, China; 3 Department of Biotechnology, Bengbu Medical College, Anhui 233030, China; 4 Department of Biochemistry and Molecular Biology, Bengbu Medical College, Anhui 233030, China

**Keywords:** Breast cancer, NT21MP, SPRY4-IT1, SKA2

## Abstract

Accumulating evidence demonstrates that long non-coding RNA (lncRNA) sprouty4-intron transcript 1 (lncRNA SPRY4-IT1) plays a vital role in the development of breast cancer. However, the underlying mechanism has not been eventually illuminated. We aimed to explore the biological activity of lncRNA SPRY4-IT1 in breast cancer cells and whether N-terminal polypeptide derived from viral macrophage inflammatory protein II (NT21MP) could exert its anti-tumor effect by regulating lncRNA SPRY4-IT1 and its target gene *SKA2*. Real-time RT-PCR, Western blotting, wound healing, and invasion assays were used to achieve this goal. We found that lncRNA SPRY4-IT1 was highly expressed in breast cancer cells. Moreover, NT21MP markedly inhibited biological effects of breast cancer cells by regulating lncRNA SPRY4-IT1, which was partially achieved through SKA2. Our findings suggested that lncRNA SPRY4-IT1 could serve as a novel biomarker by NT21MP for breast cancer.

## Introduction

Breast cancer is one of the most common malignant tumors around the world, which is a serious threat to women’s physical and mental health [1,2]. Nowadays, accumulating evidence has demonstrated that breast cancer could be mainly treated by surgery, radiotherapy, and chemotherapy [3,4]. Although in the past few years, treatments of breast cancer have made great progress, the features of cell proliferation, invasiveness, and metastasis [5,6] are still the main reason leading to higher mortality amongst breast cancer patients, which seriously affects the patients’ prognosis. However, these mechanisms are quite complex and involve many biological processes.

Long non-coding RNA (lncRNA) is a class of functional RNA molecules with a length of more than 200 nts, which do not have the ability to encode proteins. But it plays an important role in the regulation of epigenetic, transcription, and post-transcriptional levels [7]. Also many studies have suggested that lncRNA participated in breast cancer biological activity [8,9]. LncRNA sprouty4-intron transcript 1 (SPRY4-IT1), transcribed from an intron of the *SPRY4* gene, was first determined to be overexpressed in melanoma and induced cell apoptosis while depletion [10]. The ectopic expression of SPRY4-IT1 was associated with renal cell carcinoma and esophageal squamous cell carcinoma [11,12]. Sun et al. [13] found that the level of SPRY4-IT1 was positively correlated with patients’ overall survival time, while RNAi-mediated knockdown of EZH2 may induce the expression of SPRY4-IT1. Additionally, Xie et al. [14] showed that SPRY4-IT1 played an important role in epithelial–mesenchymal transition via regulating the expression of E-cadherin and vimentin. Nevertheless, the underlying mechanism of lncRNA SPRY4-IT1 in breast cancer remains unclear.

Previous studies demonstrated that N-terminal polypeptide derived from viral macrophage inflammatory protein II (NT21MP) competed effectively with CXCR4, SDF-1α, and induced cell death [15,16]. NT21MP reversed the EMT in breast cancer cells via PDGFRα [17] and exerted anti-glioma effect by specifically combining with CXCR4 [18]. In this study, we focussed on whether SPRY4-IT1 was involved in tumorigenesis and explored how NT21MP contributed to anti-tumor effects by regulating SPRY4-IT1 to provide novel biomarkers for breast cancer therapy.

## Materials and methods

### Cell culture

Human breast cancer cell lines such as SKBR-3, MCF-7, MDA-MB-231 were purchased from Shanghai Cell Institute of Chinese Academy of Science. MDA-MB-231, which overexpressed CXCR4 cell line (pcDNA-CXCR4-MDA-MB-231), was previously induced by our laboratory and has been identified. The cells were cultured in DMEM medium supplemented with 10% FBS and maintained at 37°C in a humidified atmosphere with 5% CO_2._ When cell confluence reached 80–90%, 0.25% trypsin was used for digestion and passage. All experiments were performed by using logarithmic growth phase cells.

### Total RNA extraction and quantitative real-time PCR

The total RNA of the cell lines were isolated with TRIzol (Invitrogen) according to the manufacturer’s instructions and reversed transcription into cDNA by using a Revert Aid First Strand cDNA Synthesis Kit (Thermo Scientific, U.S.A.). The quantitative real-time reverse-transcription PCR (qRT-PCR) was performed to testify the level of mRNA and in accordance with previous procedure [17]. The primers used in PCR are shown in Table 1.

**Table 1:** The sequences of primers

Name	Forward primer	Reverse primer
SPRY4-IT1	AGCCACATAAATTCAGCAGA	CAGCCTCAAGATCATCAGCA
SKA2	GTTCCAGAAAGCTGAGTCTGA	TTGCTGAATCAGGATGATTAGTC
Bak1	CCCAGGACACAGAGGAGGTTT	GCCTCCTGTTCCTGCTGATG
Bcl-2	ATGTGTGTGGAGAGCGTCAA	ACAGTTCCACAAAGGCATCC
Bax	GGGGACGAACTGGACAGTAA	CAGTTGAAGTTGCCGTCAGA
CyclinD1	AGGAGAACAAACAGATCA	TAGGACAGGAAGTTGTTG
GAPDH	CAGCCTCAAGATCATCAGCA	TGTGGTCATGAGTCCTTCCA

### Western blotting analysis

The cells were washed twice by PBS and lysed with RIPA buffer mixed with PMSF and phosphatase inhibitor cocktails (Sigma) on ice. The concentrations of protein were measured using a BCA Protein Assay Kit (Beyotime, Shanghai, China). The sample protein was fully denatured at 99°C for 10 min, and then was separated through an SDS/polyacrylamide gel and electrotransferred to PVDF membranes. The PVDF membranes were blocked in TBS Tween (TBST) containing 5% BSA for 2 h at room temperature, and then incubated with the primary antibodies overnight at 4°C. Consequently, membrane was incubated with goat anti-mouse secondary antibody (1:5000 dilution) at 37°C for 2 h. Finally, the PVDF membranes were washed three times using TBST buffer for 10 min. The blots were scanned by the Gel Image Analysis System (Bio-Rad).

### Detection of cell proliferation by SRB assay

SRB assays were performed to assess cell proliferation. Briefly, cells were seeded at 96-well culture plates before transfection. The cells were then treated with 1 µg/ml NT21MP for 24, 48, or 72 h. One hundred microliters of 0.4% SRB dye liquor was added into each well at room temperature for 30 min. The absorbance values were detected at 515 nm.

### Migration and invasion assay

The cells were seeded in six-well plates until the cells grew to 90% confluence. Wounds were created by a 10-µl pipette tip and washed with PBS. Then, some of the cells were treated with 1 µg/ml NT21MP for 24 h. Images were taken at 0 and 24 h. The invasive potential of cells was analyzed using Transwell chambers. Briefly, cells were added to the upper chamber, then cell culture medium with 10% FBS was added to the lower chamber and cultured for 20 h at 37°C. The cells on the underside were fixed with 4% paraformaldehyde and stained with Giemsa solution. The number of stained invasive cells was photographed under a microscope at 400× magnification (Olympus IX71, Tokyo, Japan). In addition, the methods being added between NT21MP and SDF-1α were as following: the cells were treated with 1 µg/ml NT21MP for 30 min at 4°C and 30 min at 37°C, then added SDF-1α (100 ng/ml, R&D Systems, Minneapolis, MN) at room temperature.

### Cell cycle and apoptosis analysis

Breast cancer cells were plated into six-well plates at a density of 1 × 10^5^ cells/well and treated with 1 µg/ml NT21MP. The cell cycle and apoptosis assay were performed as described previously (Flow Cytometry, MUSE) [17].

### Transfection of siRNA

In order to assess the lncRNA SPRY4-IT1 inhibitor, lncRNA SPRY4-IT1 siRNA were transfected in pC-MDA-MB-231 cells using Lipofectamine 2000 according to the manufacturer’s instructions (Invitrogen; Thermo Fisher Scientific, Inc.). Cells transfected with the scrambled siRNA have been adopted as the negative control. The cells were collected after 48 h transfection. Three pairs of siRNAs named siRNA SPRY4-IT1-1, siRNA SPRY4-IT1-2, and siRNA SPRY4-IT1-3. The expression level of lncRNA SPRY4-IT1 was decreased significantly by si-SPRY4-IT1-3 in comparison with the control group. Three sequences of si-SPRY4-IT1 were listed as in Table 2. The same protocol above applied to screening of SKA2-siRNA (Table 3).

**Table 2:** The sequences of lncRNA SPRY4-IT1 siRNAs

Name	Sense	Antisense
SPRY4-IT1-1	GCCCAGAAUGUUGACAGCUTT	AGCUGUCAACAUUCUGGGCTT
SPRY4-IT1-2	UCUGAUUCCAAGGCCUAUUTT	AAUAGGCCUUGGAAUCAGATT
SPRY4-IT1-3	GGGUUAUAAUAGGGAAGAUTT	AUCUUCCCUAUUAUAACCCTT

**Table 3:** The sequences of SKA2 siRNAs

Name	Sense	Antisense
SKA2-1	GGCUGGAAUAUGAAAUCAATT	UUGAUUUCAUAUUCCAGCCTT
SKA2-2	CCGCUUUAAACCAGUUGCUTT	AGCAACUGGUUUAAAGCGGTT
SKA2-3	GCAUGCACACACACACUUATT	UAAGUGUGUGUGUGCAUGCTT

### Statistical analysis

All experimental data between two different groups were performed by Student’s *t*test using GraphPad Prism 5.0 (GraphPad Software, La Jolla, CA, U.S.A.). Results are presented as the mean ± S.D. *P* values <0.05 are considered as significant.

## Results

### Effects of NT21MP and depletion or overexpression of CXCR4 on the expression of SPRY4-IT1 in breast cancer cells

**Figure 1:**
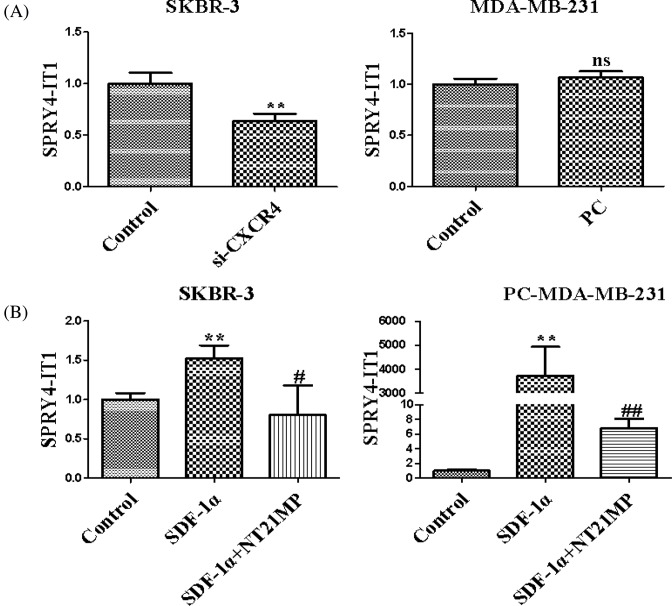
Effects of NT21MP and depletion or overexpression of CXCR4 on the expression of SPRY4-IT1 in breast cancer cells (**A**) The effects of depletion or overexpression of CXCR4 on the expression of SPRY4-IT1. (**B**) The influences of NT21MP on the expression of SPRY4-IT1. Data were presented as mean ± S.D. of three independent experiments. **P<0.05; ^#^*P* or ^##^*P*<0.05. ns represents *P*>0.05 compared with the control group.

In contrast with control group, depletion of CXCR4 could down-regulate expression of SPRY4-IT1 (Figure 1A). In the pcDNA-CXCR4 group, the expression of SPRY4-IT1 was not statistically significant compared with the control group due to low expression of SDF-1α. These results showed that the expression of SPRY4-IT1 was related to SDF-1α/CXCR4 axis. Besides, SDF-1α and NT21MP treatment were applied to further validate the role of NT21MP on SPRY4-IT1. As shown in Figure 1B, SDF-1α could promote the expression of SPRY4-IT1, while NT21MP can inhibit SDF-1α-induced up-regulation of SPRY4-IT1 expression.

### Expression of SPRY4-IT1 in breast cancer cell lines

**Figure 2:**
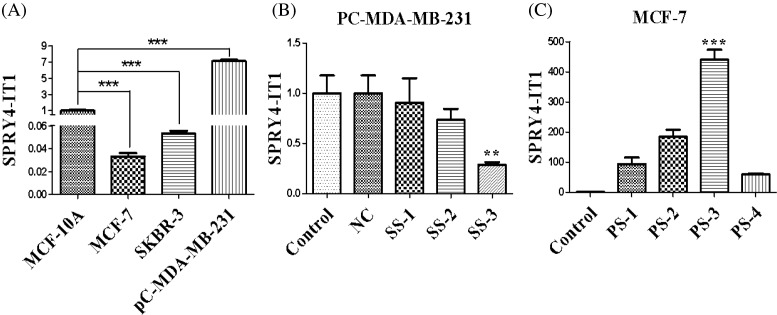
Expression of SPRY4-IT1 in breast cancer cell lines (**A**) The mRNA level of SPRY4-IT1 in breast cancer cell lines (MCF-7, pC-MDA-MB-231, and SKBR-3) was compared with human breast epithelial cells (MCF-10A). (**B**) Quantitative RT-PCR screened the best siRNAs of SPRY4-IT1 after transfection of SPRY4-IT1. (**C**) Quantitative RT-PCR screened the cell lines of overexpression of the SPRY4-IT1. The data are the result of three independent experiments and are presented as mean ± S.D.; **P<0.05; ***P<0.01. ‘PC-MDA-MB-231’ is short for ‘pcDNA-CXCR4-MDA-MB-231’, ‘SS’ is short for ‘si-SPRY4-IT1’, ‘PS’ is short for ‘pcDNA-SPRY4-IT1’.

qRT-PCR was used to investigate the mRNA level of SPRY4-IT1 in different breast cancer cell lines. Notably, the expression of SPRY4-IT1 showed the lowest expression in MCF-7 cells, while the highest in the PC-MDA-MB-231 cells compared with MCF-10A cells. Therefore, MCF-7 cells were used for SPRY4-IT1 overexpression and PC-MDA-MB-231 cells were used to interfere with SPRY4-IT1 in the following experiments (Figure 2A).

The expression level of SPRY4-IT1 after transfection with si-SPRY4-IT1 by using qRT-PCR was that si-SPRY4-IT1-3 (SS-3) showed the best effect compared with NC groups (Figure 2B). Then we selected the si-SPRY4-IT1-3 for sequencing experiments. In addition, as shown in Figure 2C, SPRY4-IT1 expression level was the highest in the pcDNA-SPRY4-IT1-3 group in comparison with NC groups. We then carried out next overexpression examination by using pcDNA-SPRY4-IT1-3 (PS-3).

### SPRY4-IT1 promotes the ability of proliferation, migration, invasion, cell cycle, and apoptosis in breast cancer cells *in vitro*

**Figure 3:**
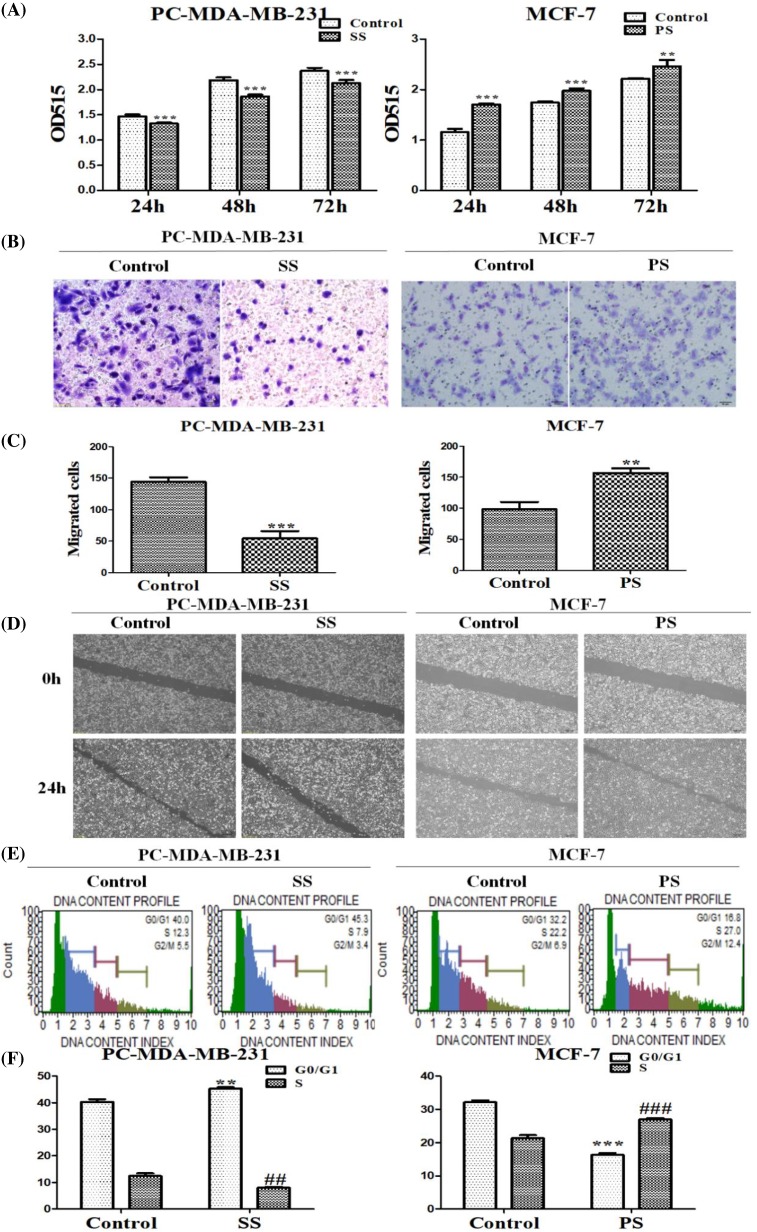

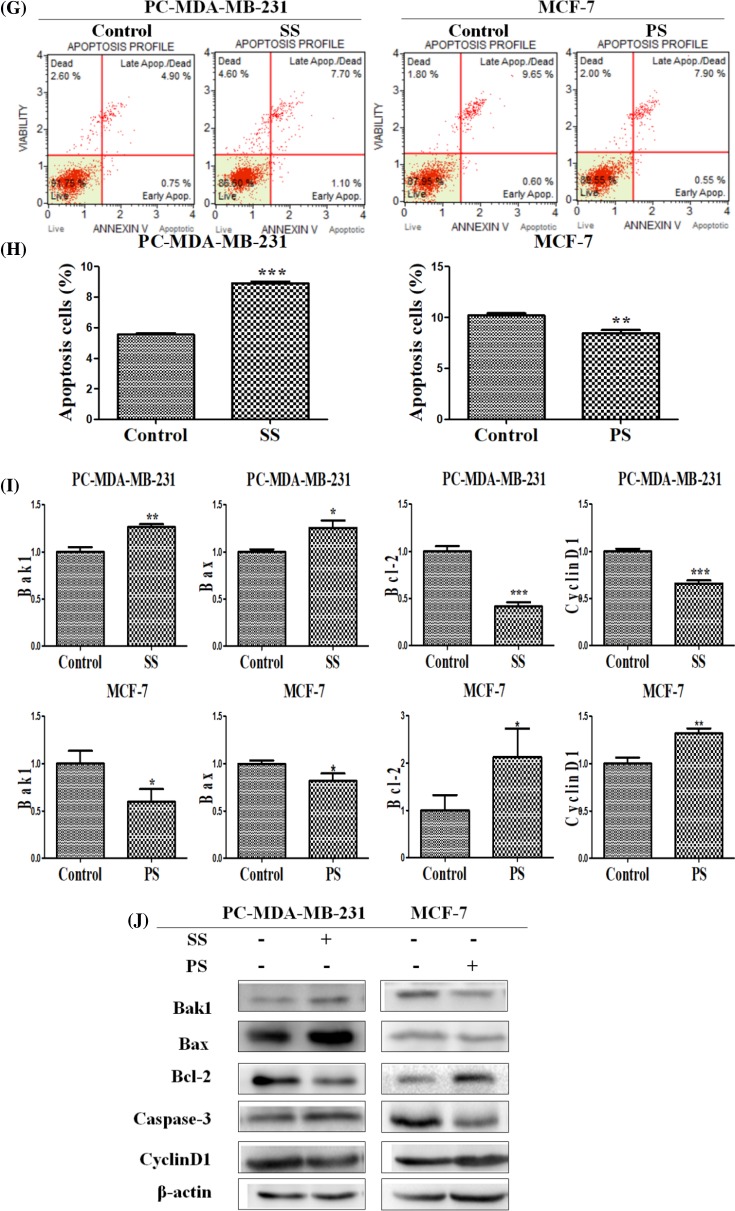
Effects of SPRY4- IT1 on cell proliferation, migration, invasion, cell cycle, and apoptosis in breast cancer cells (**A**) The influence of depletion or overexpression of SPRY4-IT1 on cell proliferation in pcDNA-CXCR4-MDA-MB-231 cells and MCF-7 cells was measured by SRB assays. (**B**) Representative images indicated the invading cells with down-regulated or up-regulated SPRY4-IT1 analyzed by Transwell assay. Scale bar: 50 μm. Data are mean ± S.D. of five fields. (**C**) Quantitative results are illustrated for (B). (**D**) The influence of depletion or overexpression of SPRY4-IT1 on cell migration in breast cancer cells was measured by wound healing assays. (**E**) Effects of SPRY4-IT1 on cell cycle in breast cancer cells. The influence of depletion or overexpression of SPRY4-IT1 on cell cycle was analyzed by propidium iodide staining and flow cytometry. Bar plots illustrating the percentage of G_0_/G_1_ and S phase in breast cancer cells with down-regulated or up-regulated SPRY4-IT1. (**F**) Quantitative results are illustrated for (E). (**G**) Effects of SPRY4-IT1 on apoptosis in breast cancer cells. The influence of depletion or overexpression of SPRY4-IT1 on cell apoptosis was evaluated by AnnexinV/PI staining and flow cytometry. (**H**) Bar plots illustrate the percentage of apoptosis cells in breast cancer cells with down-regulated or up-regulated SPRY4-IT1. (**I**) Effects of SPRY4-IT1 on cell cycle and apoptosis related factors in breast cancer cells. The influence of depletion or overexpression of SPRY4-IT1 on the mRNA level of cell cycle and apoptosis related factors was analyzed by qRT-PCR. (**J**) Western blot analyzed the protein level of cell cycle and apoptosis related factors in breast cancer cells with down-regulated or up-regulated SPRY4-IT1. Data were presented as mean ± S.D. of three independent experiments. *P or **P<0.05, ***P<0.01. ##P<0.05 or ###P<0.01 vs control group. ‘SS’ is short for ‘si-SPRY4-IT1-3’, ‘PS’ is short for ‘pcDNA-SPRY4-IT1-3’.

To evaluate the biological role of SPRY4-IT1 in the development and progression of breast cancer, we applied SRB assay to investigate the proliferation function. In Figure 3A, depletion of SPRY4-IT1 exhibited the inhibition of pcDNA-CXCR4-MDA-MB-231 compared with NC groups, whereas overexpression of SPRY4-IT1 contributed to cell proliferation in the MCF-7 cells compared with NC groups.

Transwell assays demonstrated that the invasion of cells was significantly reduced following down-regulation of SPRY4-IT1 expression and increased following up-regulation of SPRY4-IT1 expression (Figure 3B,C). Meanwhile, the influence of depletion or overexpression of SPRY4-IT1 on cell migration in breast cancer cells was measured by wound healing assays. As presented in Figure 3D, cells underwent a slower closing of scratch wound after SPRY4-IT1 knockdown in pcDNA-CXCR4-MDA-MB-231 cells compared with NC groups, while the wound healing was faster and narrower in MCF-7 cells. These findings showed that SPRY4-IT1 may be closely related to cell proliferation, migration, and invasion in breast cancer cells.

In order to explore potential phenotype change of SPRY4-IT1, we then carried out cell cycle and apoptotic assays. As shown in Figure 3E,F, the percentage of cells was markedly enhanced at G_0_/G_1_ phase (40–45.3%) and reduced at S phase (12.3–7.9%) with down-regulation of SPRY4-IT1, while the percentage of cells was markedly reduced at G_0_/G_1_ phase (32.2–16.8%) and enhanced at S phase (22.2–27%) in comparison with control group. These results indicated that SPRY4-IT1 knockdown contributed to significantly increasing proportion of apoptotic cells (5.56–8.91%), while up-regulation of SPRY4-IT1 expression contributed to decreasing proportion of apoptotic cells (10.22–8.46%) (Figure 3G,H). On the other hand, we analyzed mRNA and protein level of cell cycle and apoptosis-related genes. Compared with control group, apoptotic related genes (Bak1, Caspase3) were increased, whereas CyclinD1 and the ratio of Bcl-2/Bax were decreased due to SPRY4-IT1 depletion, while overexpression of SPRY4-IT1 caused the opposite effect. (Figure 3I,J).

### Biological effects on NT21MP combined with SPRY4-IT1

**Figure 4:**
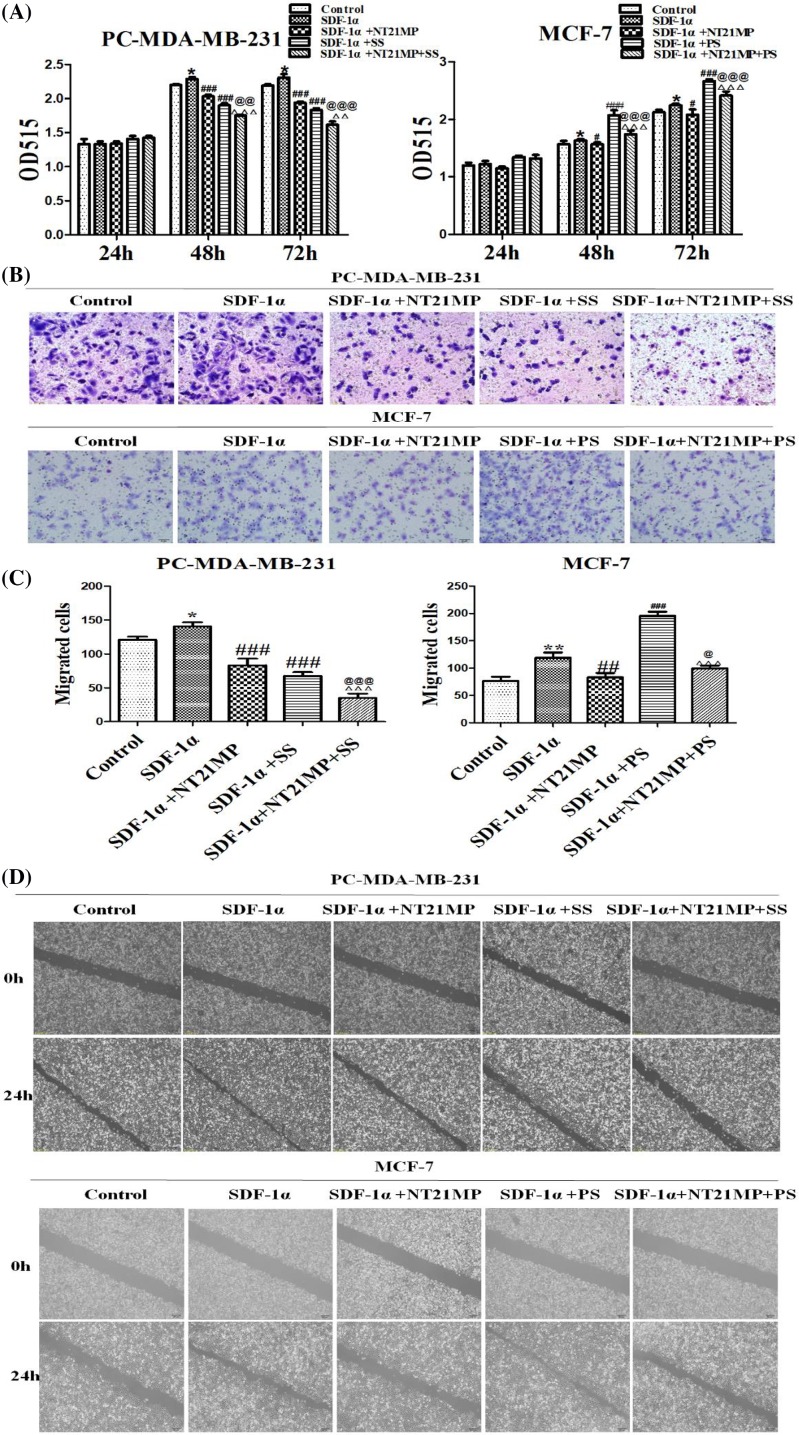

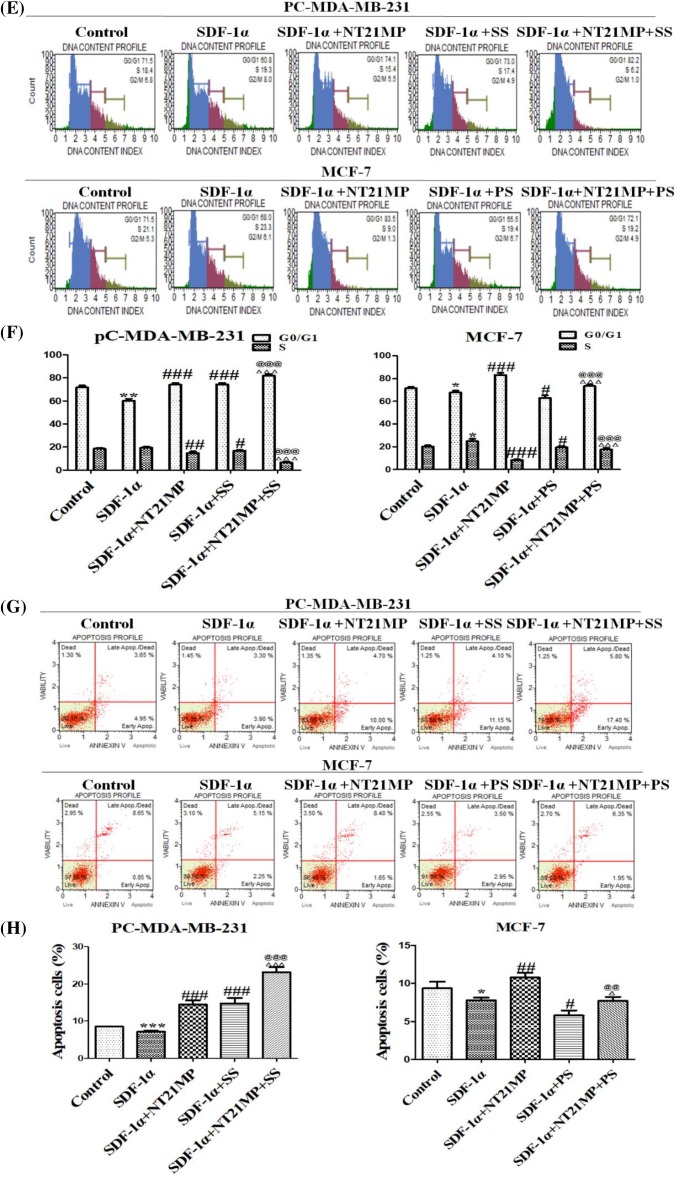
Biological effects on NT21MP combined with SPRY4-IT1 (**A**) Effects of NT21MP and depletion or overexpression of SPRY4-IT1 on cell proliferation in breast cancer cells. The cells were transfected with SPRY4-IT1-specific siRNA or SPRY4-IT1-specific pcDNA-vector and stimulated with (+SDF-1α) or not (−SDF-1α) with 100 ng/ml of SDF-1α and NT21MP (1.0 μg/ml). (**B**) Representative images indicated the invading cells with down-regulated or up-regulated SPRY4-IT1 combinded with NT21MP analyzed by Transwell assay; scale bar: 50 μm. (**C**) Quantitative results are illustrated for (B). (**D**) The influence of NT21MP and depletion or overexpression of SPRY4-IT1 on cell migration and invasion were measured by wound healing assays. (**E**) Effects of NT21MP and depletion or overexpression of SPRY4-IT1 on cell cycle in breast cancer cells. The cells were transfected with SPRY4-IT1-specific siRNA or SPRY4-IT1-specific pcDNA-vertor and stimulated with (+SDF-1α) or not (−SDF-1α) with 100 ng/ml of SDF-1α and NT21MP (1.0 μg/ml). The influence of NT21MP and depletion or overexpression of SPRY4-IT1 on cell cycle were analyzed by propidium iodide staining and flow cytometry. Bar plots illustrating the percentage of G_0_/G_1_ and S phase in breast cancer cells with down-regulated or up-regulated SPRY4-IT1 combined with NT21MP. (**F**) Quantitative results are illustrated for (E). (**G**) Effects of NT21MP and depletion or overexpression of SPRY4-IT1 on apoptosis in breast cancer cells. The influence of NT21MP and depletion or overexpression of SPRY4-IT1 on cell apoptosis were evaluated by AnnexinV/PI staining and flow cytometry. Bar plots illustrating the percentage of apoptosis cells in breast cancer cells with down-regulated or up-regulated SPRY4-IT1 combined with NT21MP. (**H**) Quantitative results are illustrated for (G). Data were presented as mean ± S.D. of five fields, *P or **P<0.05; ***P<0.01; ^#^*P* or ^##^*P*<0.05; ^###^*P*<0.01; ^@^*P* or ^@@^*P*<0.05; ^@@@^*P*<0.01; ^Δ^*P* or ^ΔΔ^*P*<0.05; ^ΔΔΔ^*P*<0.01. ‘SS’ is short for ‘si-SPRY4-IT1-3’, ‘PS’ is short for ‘pcDNA-SPRY4-IT1-3’.

The biological effects were assessed by SRB assay. Results showed that compared with the blank control group, SDF-1α promoted cell proliferation; compared with the SDF-1α group, the proliferation of NT21MP treatment group and si-SPRY4-IT1 group were inhibited, which significantly associated with the combined effect. The effect was promoted in pcDNA-SPRY4-IT1 group while higher than NT21MP group when combined with NT21MP and lower than overexpression group (Figure 4A).

The wound healing assay and Transwell assay demonstrated that cells with SDF-1α underwent a faster closing of scratch wounds and a higher invasion compared with negative control groups, NT21MP group and si-SPRY4-IT1 group showed a slower closing of scratch wounds and a lower invasion compared with SDF-1α group, especially in combined groups. In pcDNA-SPRY4-IT1 groups, the wound healing was faster and the invasion of cells was significantly increased, while the effects were higher than NT21MP group and lower than pcDNA-SPRY4-IT1 group when combined with NT21MP (Figure 4B–D).

The data showed that SDF-1α promoted cell cycle from G_0_/G_1_ phase to S phase; cells on G_0_/G_1_ phase with NT21MP group and si-SPRY4-IT1 group were decreased in S phase, particularly in combined groups. However, the percentage of cells in G_0_/G_1_ phase was significantly decreased while enhanced in S phase in pcDNA-SPRY4-IT1 groups; combined effects in G_0_/G_1_ phase resulted in lower than NT21MP groups but higher than pcDNA-SPRY4-IT1 groups (Figure 4E,F).

The results of apoptosis assays demonstrated that the percentage of apoptotic cells was inhibited by SDF-1α, while increased in NT21MP group and si-SPRY4-IT1 group, particularly in combined groups. pcDNA-SPRY4-IT1 group performed a decreasing percentage, while combined effects showed higher than pcDNA-SPRY4-IT1 group and lower than NT21MP group (Figure 4G,H).

### Influence of depletion or overexpression of SPRY4-IT1 on the SKA2

**Figure 5:**
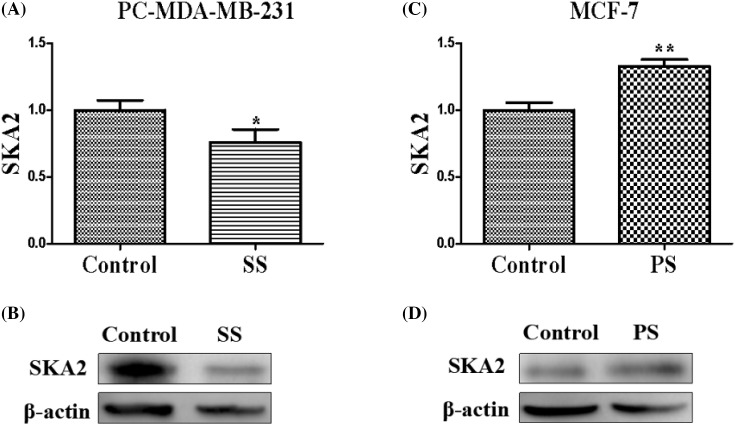
Influence of depletion or overexpression of SPRY4-IT1 on the SKA2 The influence of depletion (**A,B**) or overexpression (**C,D**) of SPRY4-IT1 on the expression of SKA2 in breast cancer cells by quantitative RT-PCR and protein level. Data were presented as mean ± S.D. of three independent experiments, *P or **P<0.05. ‘SS’ is short for ‘si-SPRY4-IT1-3’, ‘PS’ is short for ‘pcDNA-SPRY4-IT1-3’.

qRT-PCR and Western blot demonstrated that pcDNA-CXCR4-MDA-MB-231 cells transfected with SPRY4-IT1-siRNA attenuated mRNA and relative protein expression levels of SKA2, whereas MCF-7 transfected with pC-DNA-SPRY4-IT1 enhanced mRNA and relative protein expression levels of SKA2 (Figure 5). In brief, SKA2 may function as a vital downstream effector of SPRY4-IT1, which potentially mediates its effects on breast cancer tumorigenesis.

### The expression level of SKA2 in breast cancer cells

**Figure 6:**
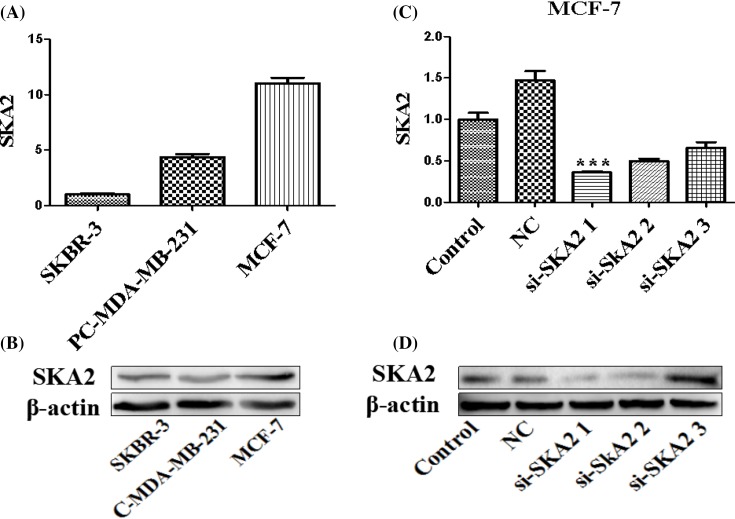
The expression of SKA2 in three breast cancer cells and the screening of the best siRNA of SKA2 (**A**) The mRNA and protein level of SKA2 in the MCF-7, pC-MDA-MB-231, and SKBR-3 cells. (**B**) The protein level of SKA2 in the MCF-7, pC-MDA-MB-231, and SKBR-3 cells. (**C**) Quantitative RT-PCR screened the best siRNAs of SKA2 after transfection of SKA2-specific siRNA or a non-targetting control siRNA for 24 h. (**D**) Western blot screened the best siRNAs of SKA2 after transfection with SKA2-specific siRNA or a non-targetting control siRNA for 24 h. Data were presented as mean ± S.D. of three independent experiments, ***P<0.01.

To further validate the relationship between SPRY4-IT1 and SKA2 on biological activity in breast cancer cells, we examined SKA2 levels (Figure 6A,B) and screened the most effective siRNAs of SKA2 after transfection with SKA2-specific siRNA or a non-targetting control siRNA for 24 h (Figure 6C,D). We found that SKA2 showed the highest expression in the MCF-7 cells and si-SKA2-1 exhibited the best effect. Thus, we selected MCF-7 cells and si-SKA2-1 for sequencing examination.

### The influence on depletion of SKA2 on biological effects in breast cancer cells

**Figure 7:**
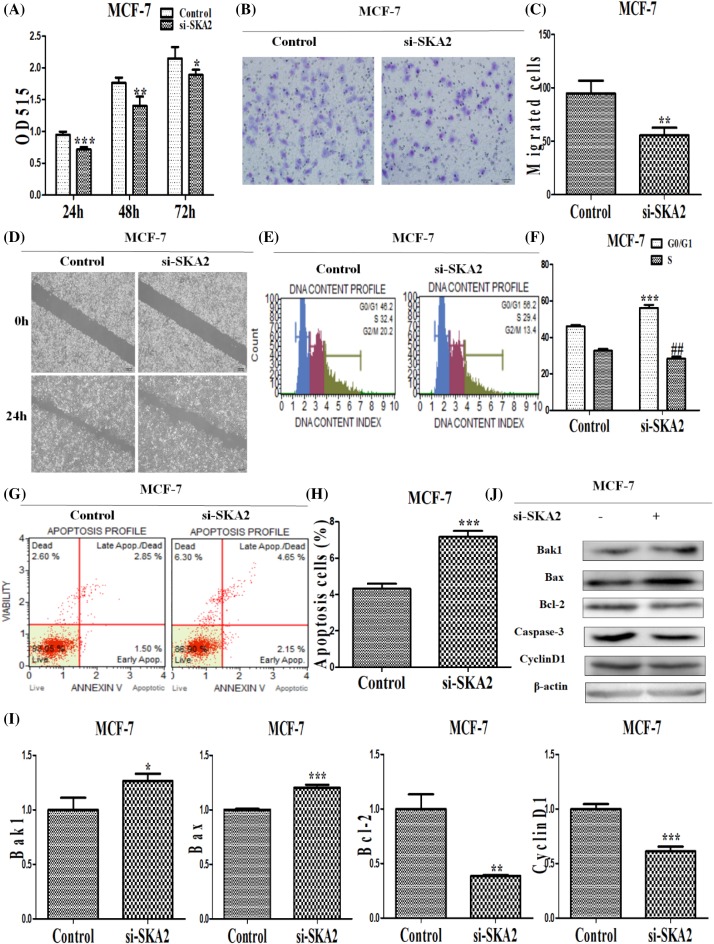
The influence of depletion of SKA2 on biological effects in breast cancer cells (**A**) The influence of depletion of SKA2 on the ability of cell proliferation in breast cancer cells. (**B**) Representative images indicated that the invading cells with down-regulated SKA2 analyzed by Transwell assays; scale bar: 50 μm. (**C**) Quantitative results are illustrated for (B). (**D**) The influence of depletion of SKA2 on cell migration and invasion was measured by wound healing assays. (**E**) The influence of depletion of SKA2 on the cell cycle in breast cancer cells. The influence of depletion of SKA2 on cell cycle was analyzed by propidium iodide staining and flow cytometry. Bar plots illustrating the percentage of G_0_/G_1_ and S phase in breast cancer cells with depletion of SKA2. (**F**) Quantitative results are illustrated for (E). (**G**) The influence of depletion of SKA2 on apoptosis in breast cancer cells. The influence of depletion of SKA2 on cell apoptosis was evaluated by AnnexinV/PI staining and flow cytometry. (**H**) Bar plots illustrating the percentage of apoptosis cells in breast cancer cells with depletion of SKA2. (**I**) Effects of depletion of SKA2 on cell cycle and apoptosis related factors in breast cancer cells. The influence of depletion of SKA2 on the mRNA level of cell cycle and apoptosis-related factors were analyzed by qRT-PCR. (**J**) Western blot analyzed the protein level of cell cycle and apoptosis-related factors in breast cancer cells with depletion of SKA2. The data are the results of three independent experiments and are presented as mean ± S.D. *P and ##*P*<0.05; **P<0.05 or ***P<0.01. ‘si-SKA2’ is short for ‘si-SKA2-1’.

To explore the function of SKA2 in breast cancer cells, we first assessed SKA2 proliferation using SRB assays and found that SKA2 knockdown inhibited cell proliferation (Figure 7A). Next, wound healing assays were performed on a slower closing of scratch wound, and the invasion of cells was significantly reduced following down-regulation of SKA2 expression by Transwell assays compared with control groups (Figure 7B–D). These findings indicated that SKA2 promotes breast cancer cells invasion and metastasis. Additionally, the results on cell cycle identified that SKA2 depletion contributed to cell cycle arresting at G_0_/G_1_ phase, increasing its proportion (46.07–56.23%) and decreasing propotion (32.7–28.4%) at S phase (Figure 7E,F). In addition, the percentage of apoptotic cells was enhanced in the treated group compared with the negative control group (Figure 7G,H). Meanwhile, as shown in Figure 7I,J, depletion of SKA2 on the mRNA level resulted in up-regulation of cell cycle and apoptosis-related factors (Bak1, Caspase3) whereas down-regulation of CyclinD1 and the ratio of Bcl-2/Bax.

### SPRY4-IT1 exhibits its function through SKA2 on biological effects in breast cancer cells

**Figure 8:**
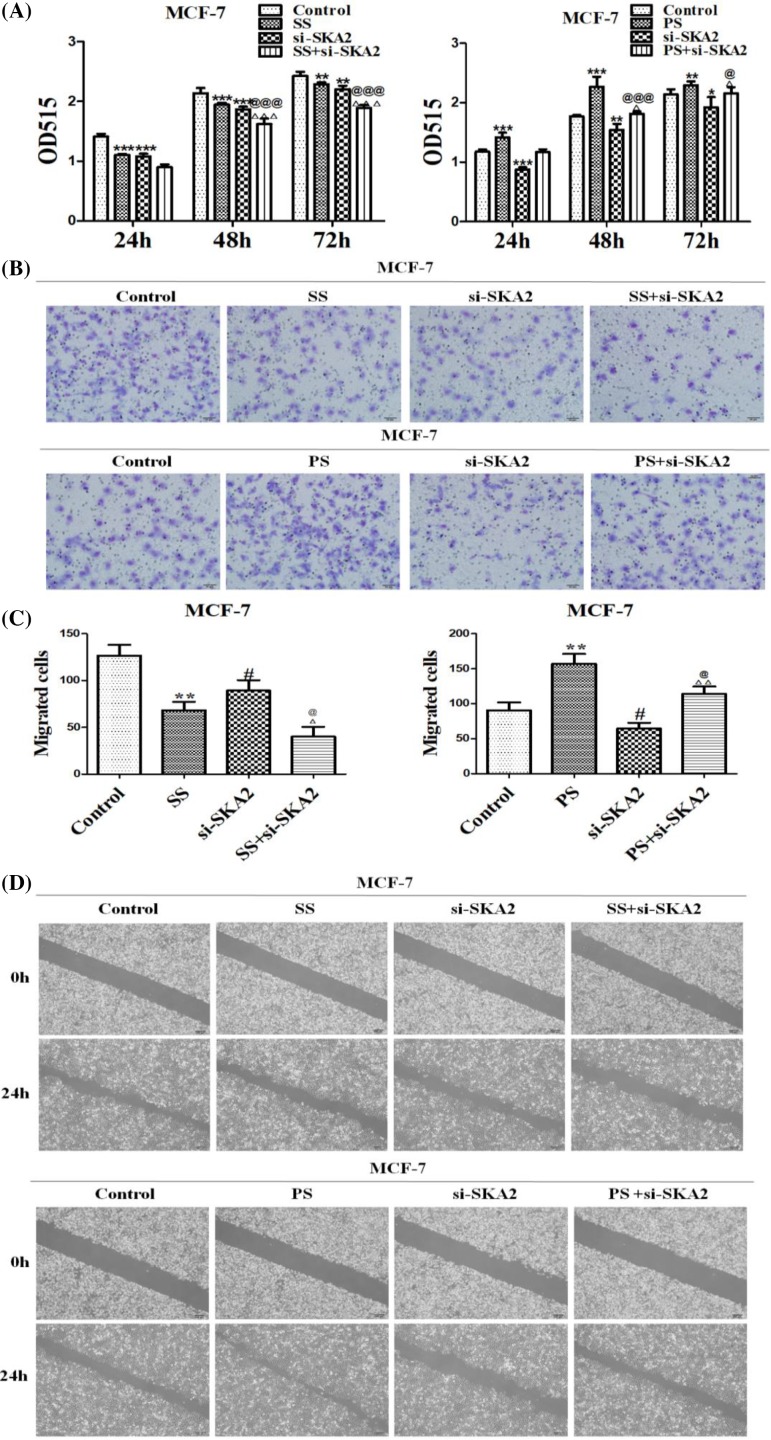

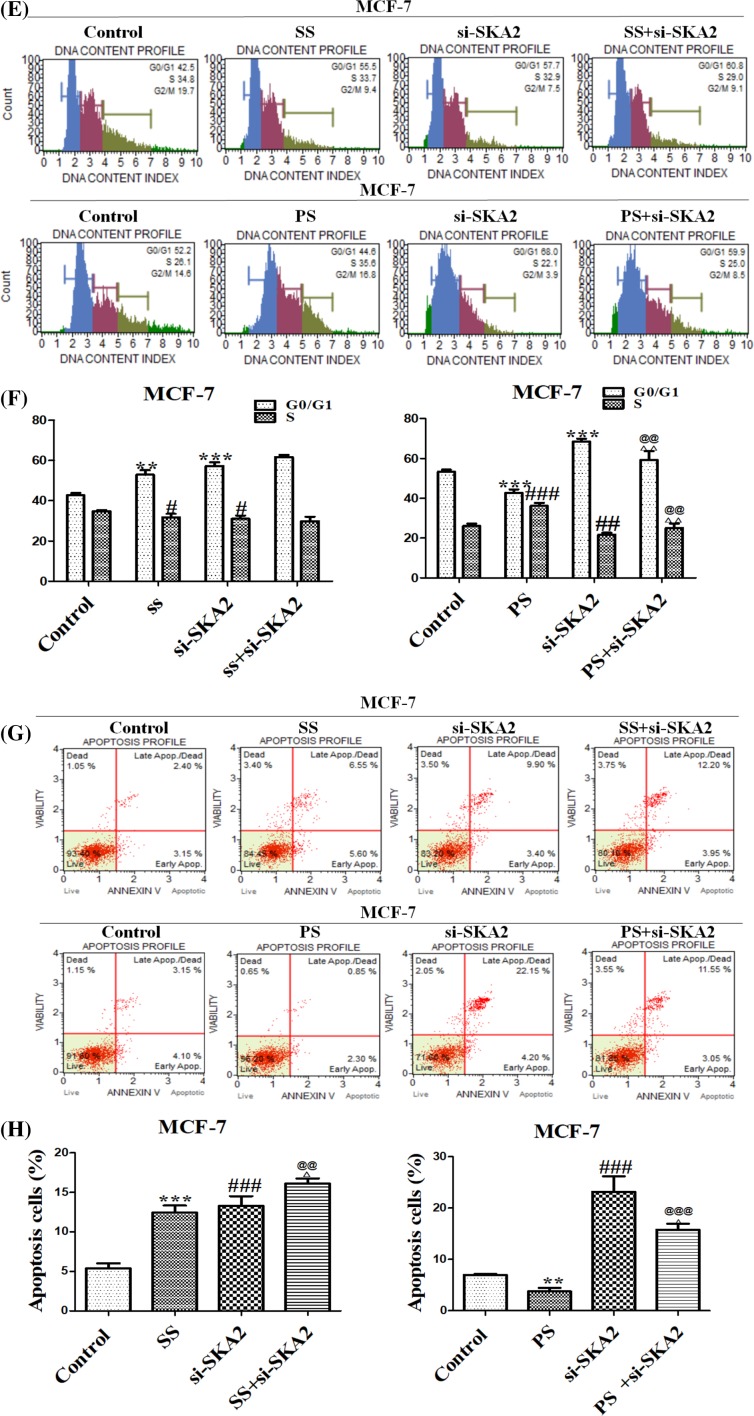
SPRY4-IT1 exhibits its function through SKA2 on biological effects in breast cancer cells (**A**) Effects of depletion of SKA2 and depletion or overexpression of SPRY4-IT1 on cell proliferation in breast cancer cells. (**B**) Representative images indicated that the invading cells with depletion of SKA2 and depletion or overexpression of SPRY4-IT1 analyzed by Transwell assay; scale bar: 50 μm. (**C**) Quantitative results are illustrated for (B). (**D**) Effects of depletion of SKA2 and depletion or overexpression of SPRY4-IT1 on cell migration and invasion in breast cancer cells were measured by wound healing assays. (**E**) Effects of depletion of SKA2 and depletion or overexpression of SPRY4-IT1 on cell cycle in breast cancer cells. The influence of depletion of SKA2 and depletion or overexpression of SPRY4-IT1 on cell cycle were analyzed by propidium iodide staining and flow cytometry. (**F**) Bar plots illustrating the percentage of G_0_/G_1_ and S phase in breast cancer cells with depletion of SKA2 and depletion or overexpression of SPRY4-IT1. (**G**) Effects of depletion of SKA2 and depletion or overexpression of SPRY4-IT1 on apoptosis in breast cancer cells. The influence of depletion of SKA2 and depletion or overexpression of SPRY4-IT1 on cell apoptosis were evaluated by Annexin V/PI staining and flow cytometry. (**H**) Bar plots illustrating the percentage of apoptosis cells in breast cancer cells with depletion of SKA2 and depletion or overexpression of SPRY4-IT1. Data were presented as mean ± S.D. of three independent experiments, **P<0.05, ***P<0.01, ^#^*P* or ^##^*P*<0.05, ^###^*P*<0.01. *P<0.05; @*P* or @@*P*<0.05; @@@*P*<0.01; Δ*P* or ΔΔ*P*<0.05,ΔΔΔ; *P*<0.01. ‘SS’ is short for ‘si-SPRY4-IT1-3’, ‘PS’ is short for ‘pcDNA-SPRY4-IT1-3’, ‘si-SKA2’ is short for ‘si-SKA2-1’.

To explore whether SKA2 participated in SPRY4-IT1 induced biological effects in breast cancer cells, MCF-7 cells were co-transfected with si-SKA2 after si-SPRY4-IT1 or pcDNA-SPRY4-IT1 transfection. First, we identified that effects of cell proliferation after transfection with si-SPRY4-IT1 or si-SKA2 were all decreased, especially co-transfection groups (Figure 8A). However, the samples that were co-transfected with pcDNA-SPRY4-IT1 and si-SKA2 had lower proliferation ability than pcDNA-SPRY4-IT1 group, but higher than si-SKA2 group. Second, we examined their function on cell migration and invasion by using wound healing and Transwell assays. We also found slower closing of scratch wounds and lower invasiveness when treated with si-SPRY4-IT1 or si-SKA2, especially co-transfection groups. Certainly, the results for co-transfection with pcDNA-SPRY4-IT1 and si-SKA2 were between pcDNA-SPRY4-IT1 group and si-SKA2 group in comparison with control groups (Figure 8B–D). Subsequently, the experimental results for cell cycle and apoptosis were significantly consistent with the above description (Figure 8E–H). Our results revealed that the effect of SPRY4-IT1 on breast cancer cells is at least partially through targetting SKA2.

### Correlation between NT21MP and SKA2

**Figure 9:**
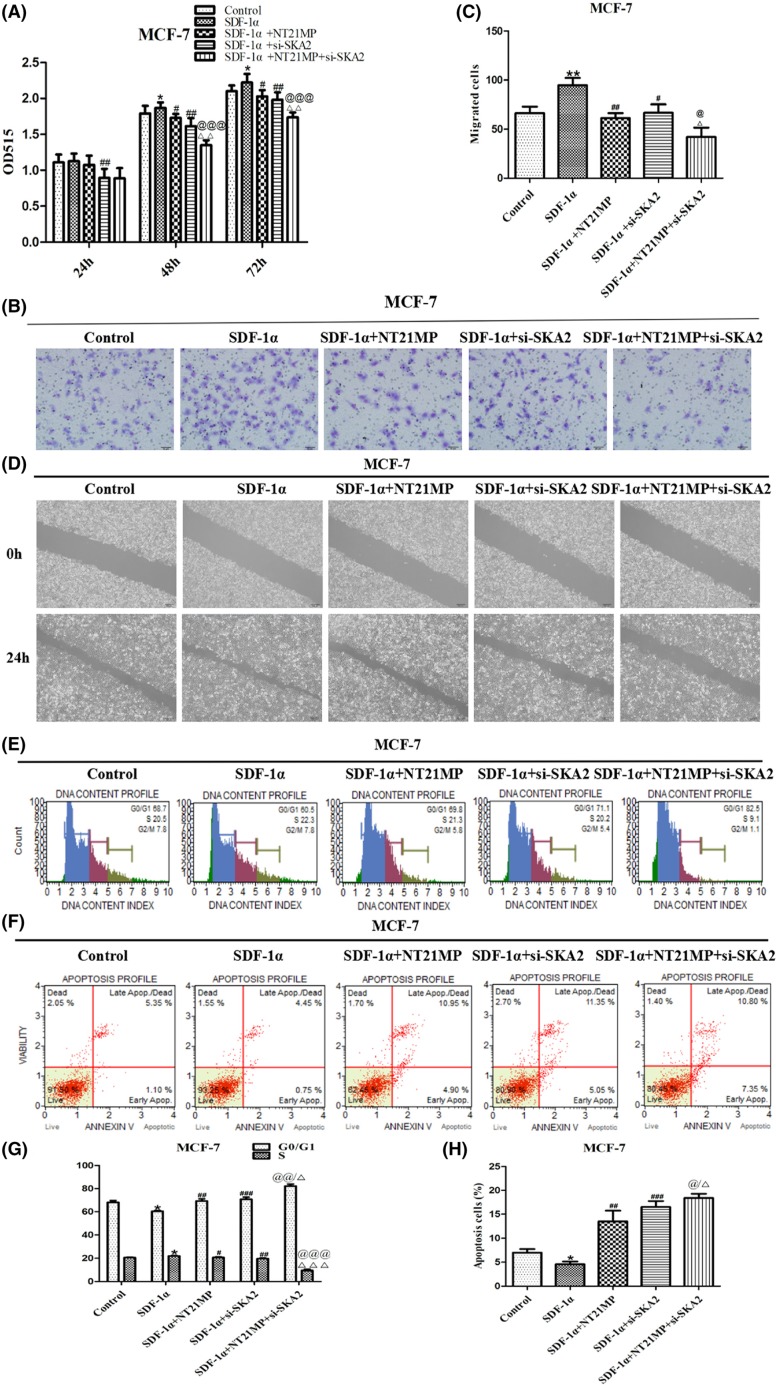
Correlation between NT21MP and SKA2 (**A**) Effects of NT21MP and depletion of SKA2 on the ability of cell proliferation in breast cancer cells. The cells were transfected with SKA2-specific siRNA and stimulated with (+SDF-1α) or not (−SDF-1α) with 100 ng/ml of SDF-1α and NT21MP (1.0 μg/ml). (**B**) The influence of NT21MP and depletion of SKA2 on cell migration and invasion were measured by Transwell assays. Representative images indicated that the invading cells with depletion of SKA2 combined with NT21MP analyzed by Transwell assay; scale bar: 50 μm. (**C**) Quantitative results are illustrated for (B). (**D**) The influence of NT21MP and depletion of SKA2 on cell migration and invasion were measured by wound healing assays. (**E**) Effects of NT21MP and depletion of SKA2 on cell cycle in breast cancer cells. The cells were transfected with SKA2-specific siRNA and stimulated with (+SDF-1α) or not (−SDF-1α) with 100 ng/ml of SDF-1α and NT21MP (1.0 μg/ml). The influence of NT21MP and depletion of SKA2 on cell cycle were analyzed by propidium iodide staining and flow cytometry. (**F**) Effects of NT21MP and depletion of SKA2 on apoptosis in breast cancer cells. The influence of NT21MP and depletion of SKA2 on cell apoptosis were evaluated by AnnexinV/PI staining and flow cytometry. (**G**) Bar plots illustrating the percentage of G_0_/G_1_ and S phase in breast cancer cells with depletion of SKA2 combined with NT21MP. (**H**) Bar plots illustrating the percentage of apoptosis cells in breast cancer cells with depletion of SKA2 combined with NT21MP. Data were presented as mean ± S.D. of three independent experiments. *P or **P<0.05; #*P* or ##*P*<0.05, ###*P*<0.01; @*P* or @@*P*<0.05, @@@*P*<0.01; △*P* or △△*P*<0.05, △△△*P*<0.01. ‘si-SKA2’ is short for ‘si-SKA2-1’.

As seen in Figure 9A, in contrast with relative group, SDF-1α promoted cell proliferation, whereas inhibition on NT21MP group or si-SKA2 group. Combined effect on si-SKA2 and NT21MP showed more obvious inhibition effect. We next examined the effects of cell migration and invasion and identified that combined effect showed slower closing of scratch wounds and lower invasiveness (Figure 9B–D). Besides, the proportion of cells at G_0_/G_1_ phase was markedly increased and decreased at S phase treated with si-SKA2 and NT21MP compared with other treatment groups (Figure 9E,G). Meanwhile, the corresponding results about cell apoptosis were in accordance with above experiments (Figure 9F,H). Our experiments indicated that the inhibition role of NT21MP in breast cancer is partially achieved through SKA2.

## Discussion

Breast cancer is one of the malignant tumors with high morbidity and mortality in the world [19]. It seriously threatens women’s physical and mental health. At present, the treatment of breast cancer is mainly based on surgery, supplemented by radiotherapy and chemotherapy [20–22]. However, the main reason causing death because of breast cancer is the high recurrence and metastasis [23]. Therefore, it is of great significance to study the ability of breast cancer cell invasion and metastasis in order to find the target of blocking tumor cell growth and metastasis.

LncRNAs were initially thought to be transcription garbage without regulating cell biological activity, but with the wide application of the two generation sequencing technology, more and more studies have shown that tumor formation was not only correlative with the protein-encoding gene but also with many lncRNAs [24–26]. LncRNA, which is more than 200 nts and not encoding protein, identified as non-protein coding RNA, are transcripts more than 200 nts in length, was found as signal molecule, decoy molecule, leader molecule, and skeleton molecule participating in gene expression and play a critical role in the regulation of the proliferation and differentiation and metastasis process of tumor cells [27–29]. Although rapid development of lncRNA formation and its role in the occurrence and development of tumor has been proved, there are still plenty of questions that need to be studied. In our previous study, we examined the expression level of lncRNAs by SDF-1α and NT21MP treatment and found that SPRY4-IT1 showed a significant change. So we selected SPRY4-IT1 for sequencing research. Accumulating evidence has suggested that SPRY4-IT1 was notably involved in carcinogenesis. It has been reported that up-regulation of SPRY4-IT1 expression promoted the migration of esophageal squamous cell carcinoma by inducing EMT [30]. Moreover, Zhou et al. [31] reported that overexpression of SPRY4-IT1 in hepatocellular carcinoma cells resulted in the proliferation and migration of cancer cells by activating EZH2. Although these data, the specific mechanism about SPRY4-IT1 ought to be further explored.

In our study, we confirmed that SPRY4-IT1 can promote proliferative, migratory, and invasive ability of cells *in vitro*, inducing transformation of cell cycle from G_0_/G_1_ phase to S phase and inhibition of cell apoptosis, which played an important role in tumorigenesis. CXCR4 has been reported to be overexpressed in breast cancer, prostate cancer, glioma, cervical cancer, lung cancer, and colorectal carcinoma [32–34]. CXCR4 regulated cell proliferation, migration, and apoptosis by the activation of PI3K/AKT, JAK/STAT, ERK signaling pathways while binding with its ligand SDF-1α [35–37]. In the previous study, we determined that NT21MP exerted its anti-tumor role by selectively blocking CXCR4 signaling pathways [38,39] and the expression level of SDF-1α is low in MDA-MB-231 cell, which is the only ligand of CXCR4. Thus, SDF-1α and NT21MP exhibited function in MDA-MB-231, which only overexpressed CXCR4 [17]. Subsequently, we adopted combination effect between NT21MP and lncRNA in order to explore whether SPRY4-IT1 was involved in NT21MP anti-tumor behavior. Our research showed that NT21MP may inhibit breast cancer cell proliferation, invasion, and migration by blocking expression levels of SPRY4-IT1. Additionally, we will carry out *in vivo* study in order to further explore the molecular activity of SPRY4-IT1, which involved in NT21MP anti-tumor activity.

**Figure 10:**
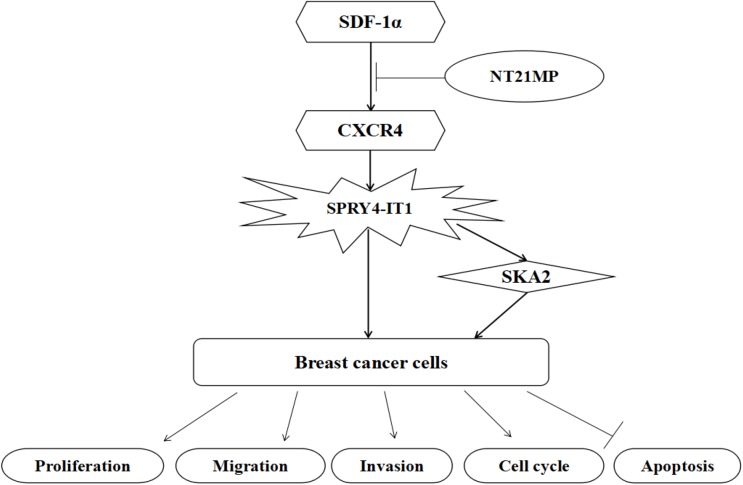
A model for SPRY4-IT1 in breast cancer cells is proposed

Accumulating evidence has demonstrated that SKA2 participated in cell cycle regulation and tumorigenesis. Cao et al. [40] reported that the expression of SKA2 and miR-301 may inhibit colony forming in A549 cells. In the present study, we examined the level amongst SKA2, SPRY4-IT1, and NT21MP, confirming *SKA2* was the target gene of SPRY4-IT1, and the regulation of SPRY4-IT1 on biological activity in breast cancer cells was partially achieved through SKA2. At the same time, SKA2 may take part in NT21MP, which regulates tumor biological activity. Although we have demonstrated NT21MP can exert its anti-breast cancer effect by regulating SPRY4-IT1 and SKA2, the specific mechanism has not been further studied. Taken together, our findings presented that NT21MP can regulate expression level of SPRY4-IT1 by blocking SDF-1α/CXCR4 axis and subsequently, activating SKA2 and playing a key role in breast cancer cell apoptosis (Figure 10). These results suggest that SPRY4-IT1 could be a promising biomarker for clinical chemotherapy.

## Conclusion

The present study demonstrated that lncRNA SPRY4-IT1 promoted breast cancer cell biological activity, whereas NT21MP could inhibit its effect by SDF-1α/CXCR4 pathway, which was partially through SKA2. Our findings indicated that lncRNA SPRY4-IT1 could serve as a novel biomarker by NT21MP for breast cancer.

## Abbreviations

CXCR4: CXC chemokine receptor 4
EMT: epithelial-to-mesenchymal transition
lncRNA: long non-coding RNA
lncRNA SPRY4-IT1: lncRNA sprouty4-intron transcript 1
NT21MP: N-terminal polypeptide derived from viral macrophage inflammatory protein II
SPRY4-IT1: sprouty4-intron transcript 1
TBST: TBS tween
SDF-1α: stromal cell-derived factor 1-alpha
